# Cerium chloride pretreatment reduces initial biofilm attachment on hydroxyapatite: a scanning electron microscopy study

**DOI:** 10.3389/froh.2025.1734138

**Published:** 2025-12-10

**Authors:** Nils Gade, Konstantin J. Scholz, Louis Kopp, Andreas Rosendahl, Wolfgang Buchalla, Annette Wiegand, Áine M. Lennon

**Affiliations:** 1 Department of Conservative Dentistry and Periodontology, University Hospital Regensburg, Regensburg, Germany; 2 Department of Preventive Dentistry, Periodontology and Cariology, University Medical Center Göttingen, Göttingen, Germany; 3 Department of Operative Dentistry and Periodontology, Center for Dental Medicine, Faculty of Medicine, Medical Center, University of Freiburg, Freiburg, Germany

**Keywords:** SEM, EDX, cerium, caries, hydroxyapatite, biofilm

## Abstract

The incorporation of cerium instead of calcium into the crystal lattice of hydroxyapatite appears to increase the resistance of dental hard tissues to caries lesion initiation and progression. The effect on initial biofilm formation is yet unknown. The aim of this study was to assess the effect of cerium(III)chloride (50%CeCl_3_) pretreatment of hydroxyapatite (HA) discs on subsequent growth of an initial 3 species caries-biofilm. Twelve 9.5 mm diameter hydroxyapatite discs were divided into three groups (*n* = 4) and treated for 1 min with either 50% CeCl_3_, ultrapure water (Control), or 0.02% chlorhexidine gluconate (CHX) and washed twice in ultrapure water for 1 min. Samples were incubated in artificial saliva (21 °C, 120 min) for pellicle formation and then placed in an active attachment caries biofilm model comprising *Actinomyces naeslundii*, *Schaalia odontolytica*, and *Streptococcus mutans*, cultured anaerobically at 37 °C for 4 h before being fixed in 2.5% glutaraldehyde and examined using scanning electron microscopy (SEM) and energy dispersive x-ray analysis (EDX) in high-vacuum mode. SEM-micrographs at up to 50,000× showed net-like or spherical precipitates on the surface of all CeCl_3_-samples but not on the Control or CHX-samples. CeCl_3_-samples also showed signs of acid attack possibly due to the low pH (2.6) of the CeCl_3_ solution. Rods and cocci were found on all Control, but only on 2 of 4 CHX samples. On CeCl_3_ samples, only one harbored isolated cocci but no rods were observed. EDX-analyses confirmed the presence of Cerium in all CeCl_3_ samples with atomic percent (At%) ranging from 0.1 to 0.4 for areas without visible precipitates and up to 4.1 for areas with precipitates. CeCl_3_-treatment before pellicle formation results in the development of precipitates on the surface of HA and appears to have potential to inhibit initial biofilm growth on HA compared to CHX treated or untreated controls.

## Introduction

The oral cavity is a complex ecosystem in which a large number of microorganisms coexist and interact. In addition to bacteria, this microbial community includes viruses, bacteriophages and fungi (1) forming biofilms that adhere to the tooth surface (2). However, before a biofilm can attach and grow, a pellicle (3) forms on the tooth surface by binding proteins and other molecules from saliva. Early colonizers such as streptococci and actinomyces species adhere to the pellicle forming an initial dental biofilm (4, 5). Even before the term was coined (6), scanning electron microscopy of what we now call “biofilm” revealed bacteria embedded in a polysaccharide-like matrix (7). Extracellular polysaccharides produced by oral bacteria such as *S. mutans* make up an important part of biofilm in the form of dental plaque (4). Depending on their composition, biofilms can shift toward an acid-producing and acid-tolerant environment in which acidophilic pathogenic microorganisms become dominant (2). Fermentation of dietary carbohydrates by these acidophilic species leads to an increase in acidic metabolites resulting in a drop in biofilm pH. This acidic pH causes demineralization of dental hard tissue at the tooth surface and the development of caries (8). Over time, therapeutic approaches to caries have evolved from preventive to operative therapy (9). Fluoride is the most widely used and well-studied caries-preventive agent (10, 11). Despite the caries decline since the introduction of fluoride, caries remains the most prevalent condition globally, particularly in disadvantaged groups (12, 13). As fluorides in systemic and local application forms are widespread and only a significant minority actively avoids fluoride use (14, 15), there is a need to develop and investigate alternative caries preventive strategies. While some bactericidal agents such as chlorhexidine digluconate (CHX) or cetylpyridinium chloride (CPC) are highly effective against oral bacteria, there are concerns about their repeated use due to potential development of resistance and shifts in oral microbiota towards caries or periodontitis (16, 17).

Cerium is a lanthanide, a rare earth element (18). As a trivalent ion, the atomic radius of cerium III is 1.01 Å and that of divalent calcium is 1.00 Å, which means that Ce III may be capable of substituting for Ca^2+^ (18). EDX analyses showed that application of CeCl_3_ resulted in the incorporation of cerium into bovine enamel (19). The replacement of Ca^2+^ by cerium in hydroxyapatite is thought to create a more stable crystal, resulting in greater acid resistance (20). Human enamel treated with CeCl_3_ has been shown to have increased resistance to demineralization (21, 22). Bacteriostatic effects on planktonic bacteria have also been demonstrated for CeCl_3_ when added directly to the culture medium (22, 23). Cerium compounds are not currently used clinically for caries or periodontitis treatment, and available safety data are limited to animal laboratory studies (24). A recent study found no difference in cytotoxicity for cerium salts including CeCl₃ and ionic fluorides (NaF, NH_4_F) (25). To our knowledge, the effects of cerium chloride pretreatment of hydroxyapatite surfaces, rinsed off prior to biofilm formation, have not yet been tested.

The primary aim of this study was to evaluate the effect of cerium III chloride (50%CeCl_3_) pretreatment of hydroxyapatite discs on the subsequent growth of an initial 3-species caries biofilm *in vitro*. A secondary aim was to determine whether cerium remains incorporated on the hydroxyapatite surface following a brief treatment and rinse protocol.

## Materials and methods

### Sample preparation

Twelve 9.5 mm diameter hydroxyapatite discs (HA-discs) (Clarkson Chromatography Products, PA, USA) were used for the experiments. The discs were sonicated in ultrapure water 10 min, repeated three times with water changes, then sterilised by dry autoclaving (Varioklav Dampfsterilisatoren, Modell 25T, H + P Labortechnik AG, Oberschleißheim, Germany) and attached to the sterile lid of an Amsterdam Active Attachment model (AAA) device (26) so that they could be submerged in test solutions or biofilm culture medium in 24 well plates (Greiner Bio-one, Kremsmünster, Austria).

### HA-disc treatment

Twelve discs each were assigned to one of three test groups. The first group (CeCl_3_) was treated with a 50% cerium (III) chloride, CeCl_3_, solution Sigma Aldrich, St. Louis, MO, USA (pH 2.6). The second group (CHX group) was treated with 0.02% chlorhexidine gluconate (CHX), (pH 5.5) solution, prepared by diluting a 20% CHX stock (Sigma Aldrich, St. Louis, MO, USA) with ultrapure water. The third group (Control), which served as the negative control, was treated with ultrapure water only (pH 6.8). Each disc was placed in 1.5 mL of the respective treatment solution for 1 min. Thereafter samples were washed twice in ultrapure water, for 1 min each.

### Artificial saliva and pellicle formation

Artificial saliva was prepared as described by Hahnel, 2007 (27) by dissolving 850 mg mucin (Sigma Aldrich, St. Louis, MO, USA), 10 mg lysozyme, 1,000 mg alpha-amylase and 40 mg bovine albumin (Sigma Aldrich, St. Louis, MO, USA) in 1 L phosphate buffered saline (PBS). The solution was sterile filtered up to 0.2 µm (Steritop-GP Merk-Milipore, Darmstadt, Germany). An artificial pellicle was formed on each HA sample surface by incubating in 1.5 mL of artificial saliva at 21°C for 2 h.

### Bacterial strains and culture conditions

The following species were obtained as dried cultures from the German Collection of Microorganisms and Cell cultures (DMSZ, Braunschweig, Germany): *Actinomyces naeslundii* (DSM 43013), *Schaalia odontolytica* (DSM 19120), and *Streptococcus mutans* (DSM 20523)*.* Planktonic cultures of each were grown anaerobically at 37°C overnight (80% N_2_, 10% CO_2_, and 10% H_2_) in a microincubator (MI23NK, SCHOLZEN Microbiology Systems, St. Margrethen, Switzerland). Tryptic soy broth TSB (Sigma Aldrich, St. Louis, MO, USA) was used as a liquid medium for *S. mutans,* brain heart infusion (BHI) broth (Merk, Darmstadt, Germany) was used for *A. naeslundii* and *S. odontolytica*. Overnight cultures were harvested by centrifugation (ROTINA 420 R, 129 Hettich Lab Technology, Tuttlingen, Germany). Pellets were resuspended in biofilm culture medium (BCM) consisting of 10% fetal bovine serum (FBS; Gibco Life Technologies, Carlsbad, CA, United States), 40% artificial saliva as described above and 50% modified fluid Universal medium (mFUM) supplemented with 67 mmol/L Sørensen's buffer (pH 7.2), containing carbohydrate 0.15% (w/w) glucose and 0.15% (w/w) sucrose (28). The optical density (OD) for each bacterial suspension was adjusted to 0.5 at 600 nm (Ultrospec 3300; Amersham Biosciences, Amersham, UK), and the three bacterial suspensions in BCM were then mixed in equal parts, resulting in the final BCM inoculum.

### Biofilm formation

A 24-well plate was then prepared, containing 1.5 mL of BCM inoculum for polymicrobial biofilm formation in each well. Subsequently, the AAA-lid holding the HA-discs with artificial pellicle formed as described above, was placed on the 24-well plate, immersing the samples in BCM inoculum, and incubated for 4 h at 37 °C under anaerobic conditions allowing initial attachment to the samples (MI23NK, SCHOLZEN Microbiology Systems, St. Margrethen, Switzerland). Biofilm was formed and observed on the hydroxyapatite discs.

### Preparation for SEM

For this experiment, 12 samples (4 for each group) were used for the electron microscopic investigation of initial attachment and energy dispersive x-ray spectroscopy (EDX). Following biofilm formation, the samples were removed from the AAA lid and fixed in 2.5% glutaraldehyde in 0.1 M Sørensen buffer (pH 7.2) for 2 h. Then the samples were then washed twice with Sørensen buffer and three times in ultrapure water for 15 min each. Dehydration was achieved via ascending alcohol series using 30%, 50%, 70%, 80%, 90%, 96%, and 100% (v/v) graded ethanol, for 20 min each. Finally, the samples were placed in an exsiccator for drying overnight. All steps were carried out at room temperature.

Each HA-disc was mounted onto an aluminum stub (25 mm diameter) using double-sided adhesive carbon discs and conductive adhesive paste (model Leit-C-Tab and Leit-C-Plast, manufactured by Baltic Präparation e.K., Wetter, Germany). Then, the discs were sputtered with platinum (BAL-TEC SCD 005, Baltic Präparation e.K, Wetter, Germany).

### Surface visualization (HV-SEM)

All sample surfaces were visualised using scanning electron microscopy (FEI Quanta 400 FEG, Thermo Fisher Scientific, FEI Deutschland GmbH, Dreieich, Germany) in high vacuum mode with secondary electron detector Everhart-Thornley Detector ETD (accelerating voltage 3 kV, working distance 6–7 mm, Spot 3, ap. 4, tilt 30°). The entire surface was imaged at 50,000× magnification, after which representative areas were chosen for examination and imaging.

### Surface elemental composition (EDX)

Three CeCl_3_ samples and two samples each for the Control and CHX groups were further analysed with EDX to examine the elemental composition of the sample surfaces (EDAX Octane Elect Detector, APEX v2.5 AMETEK EDAX GmbH, Unterschleissheim, Germany). For each sample, three to four randomly chosen areas were analysed. Additional measurements were made in the CeCl_3_ samples of areas with and without surface precipitates. The EDX measurements were performed in high vacuum mode (acceleration voltage 10 kV, working distance 10 mm, ap 4, 100 live seconds, amp time 3.84 μs). The atomic percentages (At%) of the elements C, Ca, Ce, Cl, P, O, N, Na and Mg were calculated from each sample. The horizontal field width for EDX measurements was between 190 µm and 4.83 µm.

### Statistical analyses

Data visualisation and statistical analyses were carried out using Graphpad Prism 10, GraphPad Software, Boston, MA USA. All data points recorded are shown. Regression analysis was performed for relative atomic % Ca and relative atomic % Ce based on 24 independent EDX measurements obtained from the cerium-treated samples.

## Results

### Surface visualization (HV-SEM)

**Figure 1:**
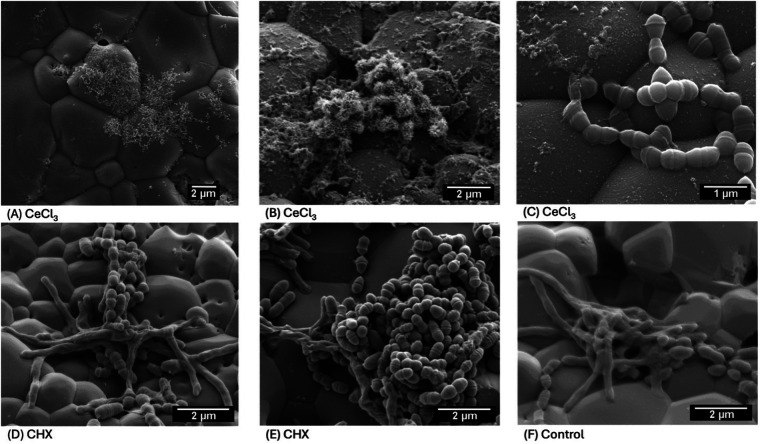
**(A)** Net-like precipitates on the surface of a CeCl_3_ sample. Magnification 15,000×. HFW 18.03 µm **(B)** net-like and spherical precipitates on the surface of a CeCl_3_ sample. Magnification 25,000×. horizontal field width (HFW) 10.82 µm. Cerium precipitates were found on the surface of all CeCl_3_ samples, confirmed by At%Ce >0 in all EDX analyses. **(C)** Isolated cocci were found on the surface of one CeCl_3_ sample, this was not representative of the CeCl_3_ samples. Some of these cocci showed morphological deformations. Magnification 50,000×. HFW 5.41 µm. **(D,E)** Rods and cocci on the surface of a sample, treated with CHX. Magnification 30,000×. HFW 9.01 µm. Rods and cocci were found on 2 of 4 CHX treated samples. **(F)** Rods and cocci on the surface of a negative control sample (ultrapure water only). Magnification 30,000×. HFW 9.01 µm. Rods and cocci were found on all negative control samples.

Figures 1A–F show the SEM-Micrographs with a cobblestone-like morphology of the sintered HA sample surfaces.

In the CeCl_3_ group, Figures 1A,B, show both flat net-like and raised spherical precipitates. These were found on all samples of the CeCl_3_ group. The spherical precipitates had a distinctive spherical core surrounded by spiked projections of 0.1 µm. (A, B) Show that the precipitates were not uniformly distributed across the sample surface, with some areas being free of precipitates. None of the samples in the CHX group or in the control group showed precipitates on their surfaces in (D–F).

Biofilm consisting of both rods and cocci was found on 2 of 4 CHX- treated, and all untreated controls. In (D, E) bacteria were found in complex multi-species arrangements sometimes reaching several cells in height. No bacteria were found on 3 of 4 CeCl_3_ treated samples. Isolated *S. mutans* were found on the surface of one CeCl_3_ sample shown in (C). No rods were found on any of the CeCl_3_ treated samples. Figure 1F shows rods and cocci on a sample from the untreated control group.

### Surface elemental composition (EDX)

**Figure 2:**
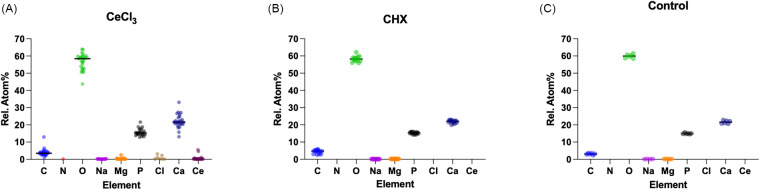
Figures **(A–C)** show the different elements and their respective relative atomic percentages (At%) measured by EDX for the three groups. Each individual measurement is shown as a single point. Median is shown as a horizontal bar.

Figures 2A–C shows the relative atomic percent (At%) for the elements carbon, nitrogen, oxygen, sodium, magnesium phosphorus, chlorine and calcium measured on the hydroxyapatite-surfaces treated according to the three groups. There was little difference in the atomic percentage of the elements between the CHX and control group. Samples in the CeCl_3_ group have more variation in At% of carbon, oxygen, phosphorus, magnesium and calcium. Cerium and chloride were found on all samples in the CeCl_3_ group, but not in the CHX or Control group.

**Figure 3:**
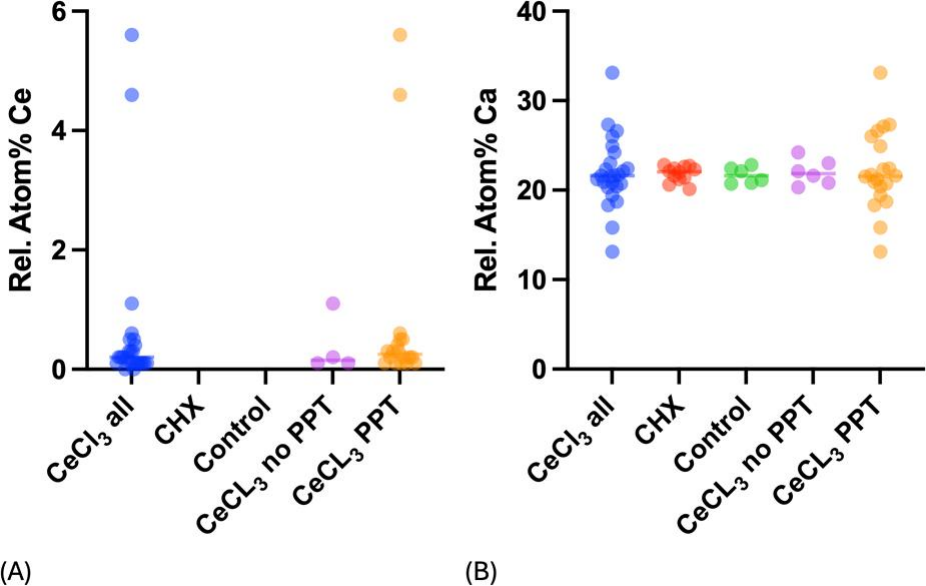
**(A,B)** Relative atomic percent (At%) of cerium and calcium in three different groups, as well as in areas with and without precipitates (PPT) in the CeCl_3_ group. Each individual measurement is shown as a single point. Median is shown as a horizontal bar.

Figures 3A,B show At% of cerium and calcium in the three test groups and also for CeCl_3_ samples in areas with and without preciptates (CeCl_3_ PPT and CeCl_3_ no PPT). The CeCl_3_ group shows a higher At% Ca variation compared to CHX treated and control group. When comparing the different specimens in the CeCl_3_ group, it is evident that the samples with precipitates exhibited the greatest At% Ca variation. The CHX and control groups showed the least At% Ca variation. The CeCl_3_ group without precipitates showed more variation in At% Ca than the CHX and control groups, but less than the samples with precipitates.

**Figure 4:**
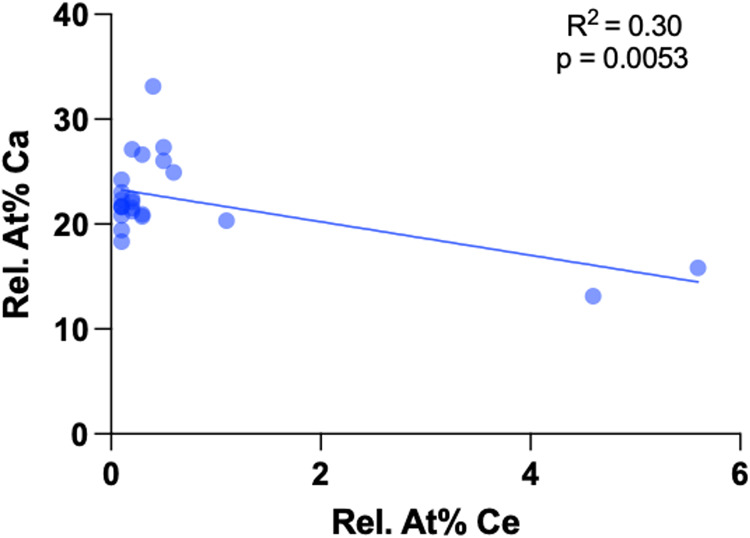
The negative slope shows a linear regression. Increasing cerium is associated with lower calcium levels in the cerium-treated samples.

Figure 4 shows a linear regression for At% Ca to At% Ce for CeCl_3_ treated samples. The linear fit shows a negative slope (*R*² = 0.30, *p* = 0.0053), indicating that increasing cerium is associated with lower calcium conten. Most of the measured points show low Ce (<1 At%), with calcium values around 20–25 At%. A few samples with higher cerium contents (up to approximately 5 At%) are characterized by lower calcium contents (12–15 At%). This underscores the trend that cerium substitution into the hydroxyapatite lattice is accompanied by a reduction in calcium content.

## Discussion

In the present study, pretreatment of HA-Discs with 50% CeCl₃ reduced initial biofilm formation compared to 0.02% CHX, or ultrapure water (control) treated samples. The 3 species caries model used here contains the cariogenic species *S. odontolytica* and *S. mutans* in addition to *A. naeslundii*, which functions as a bridging organism, facilitating co-adhesion and structural maturation in early biofilms (29, 30). This three-species model has been used previously to investigate multispecies biofilm formation (29, 31), although in this case we specifically investigated 4 h old initial bacterial colonization (32). The CHX treated and untreated controls show formation of microcolonies characteristic of initial biofilm formation.

SEM was used for qualitative assessment of surface morphology, not to generate quantitative data. As is standard in SEM-based surface analyses, reproducibility was assessed by examining multiple independent imaging fields across several HA discs per condition. Because SEM sample preparation is destructive, identical technical replicates of the same specimen are not feasible. SEM micrographs revealed a marked reduction in bacterial adhesion on the CeCl₃ treated discs and characteristic precipitates on all samples in the CeCl₃ group. These spherical precipitates have previously been described in cerium doped hydroxyapatite and on cerium treated human and bovine enamel and dentin surfaces (19, 23, 33, 34).

CHX was included as an antibacterial control for immediate surface effects. Even after rinsing, CHX exhibits significant substantivity on oral surfaces making it suitable for comparison as an antibacterial in initial-attachment assays (35, 36). Fluoride on the other hand, was not included as a control in this study because its primary mechanisms of action are enamel remineralisation, fluorapatite formation and reduction of enamel solubility, which were not tested here.

EDX analyses confirmed the presence of cerium in all CeCl₃ treated samples both in the precipitates and in areas free of precipitates, consistent with previous studies showing similar precipitations after cerium-salt application on enamel with and without pellicle or dentin (19, 23, 34). A significant negative correlation of cerium to calcium was found indicating a true lattice substitution rather than just surface adsorption.

The trivalent cerium ion and the divalent calcium ion have nearly identical ionic radii which facilitates substitution into the hydroxyapatite lattice (18). However, substituting a divalent ion with a trivalent ion leads to a positive charge imbalance, which must be compensated within the lattice, typically by permitting calcium vacancies, incorporating anions or lattice distortion (37, 38). Kaur et al. found that replacing Ca²^+^ with the lanthanide Ce³^+^ results in the replacement of OH^−^ with O²^−^, which stabilizes the lattice by decreasing the distance between ions and lowering the crystal energy (39). Predoi et al. found trivalent cerium in cerium doped hydroxyapatite next to tetravalent cerium. Surface property changes such as shifts in zeta potential or conductivity may explain reduced initial biofilm attachment to Ce doped hydroxyapatite (33, 37). Physical surface properties play a central role in initial biofilm formation by influencing protein adsorption, bacterial attachment, and binding of extracellular polymeric substances (EPS) (40). We exposed samples to CeCl_3_ for just 1 min and then rinsed twice using ultrapure water to prevent any direct bactericidal effects from residual cerium remaining in the biofilm media. In contrast, CHX treatment of samples, which were also rinsed, did not completely prevent bacterial adhesion despite its known high substantivity and bactericidal properties (17, 41).

Another antimicrobial assay showed that cerium doped hydroxyapatite inhibited the growth of colony forming units for all tested strains (33). Also, a suspension of cerium and hydroxyapatite showed a biocidal effect against E. coli and C. albicans after 72 h incubation (33). *S. mutans* is a key acidogenic and aciduric species in cariogenic biofilms. The SEM observations in our study indicate that initial attachment is reduced on CeCl_3_ treated surfaces compared to CHX treated or untreated controls. This aligns with earlier work showing that initial attachment is essential in *S. mutans* colonization and subsequent EPS dependent biofilm maturation (42, 43). Our findings suggest that CeCl_3_ treatment may disrupt *S. mutans* as an early colonizer in a multispecies biofilm. Hydrolyzed Ce(IV) salts have been shown to reduce adhesion of *S. mutans* biofilm formation by about 40% but have very little effect on planktonic growth, indicating that cerium has a specific effect on initial adhesion rather than a direct bactericidal effect on *S. mutans* (44). Similarly, catheter surfaces with a slight positive charge have been shown to inhibit biofilm formation over 60- and 120-min growth periods for different microorganisms, compared to uncharged samples (45). However, Terada et al. showed that an 8-h-old *E. coli* biofilm had 23 times higher cell density on a positively charged diethylaminoethyl methacrylate surface than on a negatively charged sodium styrene sulfonate surface (46). An *in vitro* study could show that the cell walls of both Gram-positive and Gram-negative bacteria have been shown to have a negative charge (47). In another study, Gottenbos et al. demonstrated that positively charged surfaces can inhibit the growth of bacteria, particularly Gram-negative bacteria. Negatively charged surfaces resulted in slower initial adhesion of bacteria, though they did not affect the growth of Gram-negative or Gram-positive bacteria (48). Therefore, the interaction between bacteria and the surface of cerium-enriched HA could be due to the negatively charged bacterial wall and the positively charged surface of the HA. This could explain why the initial biofilm attachment is lower in cerium-enriched HA than in HA treated with chlorhexidine (CHX) or water. Kurniawan et al. demonstrated that biofilm formations show both negative and positive parts (49). A recent study found cerium oxide nanoparticles enhanced the antibacterial activity of chlorhexidine (50). Synergy depends strongly on the specific cerium compound and formulation, these findings cannot be assumed for the soluble CeCl₃ used in the present study. It is important to examine CeCl₃ separately to clarify its own surface-related mechanisms during early biofilm formation. While testing of interactions with other antibacterials and cariostatics was beyond the scope of the present study, it is a topic that could be explored in future studies.

In the present study we examined a three-species caries biofilm. To our knowledge, the effect of CeCl₃ pretreatment of HA discs has never been tested using multispecies biofilms. Although CeCl_3_ application has been shown to result in increased surface roughness (51, 52) due to the acidic pH and subsequently more surface artifacts, less bacterial attachment was visible in the present study. Future studies should investigate whether the inhibitory effects we found also extend to more complex microcosm biofilms and in adapted lanthanide-salt containing formulations with reduced concentrations or adapted pH-values. We chose a 4-h incubation time in order to examine early biofilm attachment, but longer culture times are necessary to determine effects on mature biofilms. Combining CeCl₃ pretreatment with fluoride exposure in future studies would also more closely mimic clinical situations. Our study provides qualitative scanning electron microscopy (SEM) data. Further studies should investigate the quantitative effects on biofilm growth.

## Conclusion

Within the limitations of the study, it can be concluded that CeCl_3_ treatment before pellicle formation results in the development of precipitates on the surface of HA and appears to have potential to inhibit initial biofilm growth compared to CHX-treated or untreated controls. Further investigations are needed to identify the mechanisms involved and to test these effects under conditions closer to the clinical situation.

## Data Availability

The original contributions presented in the study are included in the article/Supplementary Material, further inquiries can be directed to the corresponding author.

## References

[1] Baker JL Bor B Agnello M Shi W He X. Ecology of the oral microbiome: beyond bacteria. Trends Microbiol. (2017) 25(5):362–74. 10.1016/j.tim.2016.12.012 28089325 PMC5687246

[2] Pitts NB Twetman S Fisher J Marsh PD. Understanding dental caries as a non-communicable disease. Br Dent J. (2021) 231(12):749–53. 10.1038/s41415-021-3775-4 34921271 PMC8683371

[3] Bowen WH Koo H. Biology of *Streptococcus mutans*-derived glucosyltransferases: role in extracellular matrix formation of cariogenic biofilms. Caries Res. (2011) 45(1):69–86. 10.1159/000324598 21346355 PMC3068567

[4] Bowen WH Burne RA Wu H Koo H. Oral biofilms: pathogens, matrix and polymicrobial interactions in microenvironments. Trends Microbiol. (2018) 26(3):229–42. 10.1016/j.tim.2017.09.008 29097091 PMC5834367

[5] Rosan B Lamont RJ. Dental plaque formation. Microbes Infect. (2000) 2(13):1599–607. 10.1016/S1286-4579(00)01316-2 11113379

[6] Mack WN Mack JP Ackerson AO. Microbial film development in a trickling filter. Microb Ecol. (1975) 2(3):215–26. 10.1007/BF02010441 24241336

[7] Jones HC Roth IL Sanders WM. Electron microscopic study of a slime layer. J Bacteriol. (1969) 99(1):316–25. 10.1128/jb.99.1.316-325.1969 5802613 PMC250005

[8] Selwitz RH Ismail AI Pitts NB. Dental caries. Lancet. (2007) 369(9555):51–9. 10.1016/S0140-6736(07)60031-2 17208642

[9] Gao SS Zhang S Mei ML Lo ECM Chu CH. Caries remineralisation and arresting effect in children by professionally applied fluoride treatment—a systematic review. BMC Oral Health. (2016) 16:12. 10.1186/s12903-016-0171-6 26831727 PMC4736084

[10] Buchalla W Attin T Schulte-Mönting J Hellwig E. Fluoride uptake, retention, and remineralization efficacy of a highly concentrated fluoride solution on enamel lesions in situ. J Dent Res. (2002) 81(5):329–33. 10.1177/154405910208100508 12097446

[11] Silverstone LM. Remineralization of human enamel in vitro. Proc R Soc Med. (1972) 65(10):906–8. 5085103 10.1177/003591577206501059 PMC1644602

[12] GBD 2021 Oral Disorders Collaborators. Trends in the global, regional, and national burden of oral conditions from 1990 to 2021: a systematic analysis for the global burden of disease study 2021. Lancet. (2025) 405(10482):897–910. 10.1016/S0140-6736(24)02811-3 40024264

[13] Kassebaum NJ Smith AGC Bernabé E Fleming TD Reynolds AE Vos T Global, regional, and national prevalence, incidence, and disability-adjusted life years for oral conditions for 195 countries, 1990–2015: a systematic analysis for the global burden of diseases, injuries, and risk factors. J Dent Res. (2017) 96(4):380–7. 10.1177/0022034517693566 28792274 PMC5912207

[14] Newbrun E. The fluoridation war: a scientific dispute or a religious argument? J Public Health Dent. (1996) 56(5):246–52. 10.1111/j.1752-7325.1996.tb02447.x 9034969

[15] Whelton HP Spencer AJ Do LG Rugg-Gunn AJ. Fluoride revolution and dental caries: evolution of policies for global use. J Dent Res. (2019) 98(8):837–46. 10.1177/0022034519843495 31282846

[16] Früh R Anderson A Cieplik F Hellwig E Wittmer A Vach K Antibiotic resistance of selected bacteria after treatment of the supragingival biofilm with subinhibitory chlorhexidine concentrations. Antibiotics (Basel). (2022) 11(10):1420. 10.3390/antibiotics11101420 36290078 PMC9598507

[17] Mao X Hiergeist A Auer DL Scholz KJ Muehler D Hiller KA Ecological effects of daily antiseptic treatment on microbial composition of saliva-grown microcosm biofilms and selection of resistant phenotypes. Front Microbiol. (2022) 13:934525. 10.3389/fmicb.2022.934525 35847089 PMC9280182

[18] Jakupec MA Unfried P Keppler BK. Pharmacological properties of cerium compounds. Rev Physiol Biochem Pharmacol. (2005) 153:101–11. doi: 10.1007/s10254-004-0024-6 15674649 10.1007/s10254-004-0024-6

[19] Scholz KJ Hiller KA Ebensberger H Ferstl G Pielnhofer F Tauböck TT Surface accumulation of cerium, self-assembling peptide, and fluoride on sound bovine enamel. Bioengineering. (2022) 9(12):760. 10.3390/bioengineering9120760 36550966 PMC9774660

[20] Wegehaupt FJ Sener B Attin T Schmidlin PR. Application of cerium chloride to improve the acid resistance of dentine. Arch Oral Biol. (2010) 55(6):441–6. 10.1016/j.archoralbio.2010.03.016 20399417

[21] Wegehaupt FJ Buchalla W Sener B Attin T Schmidlin PR. Cerium chloride reduces enamel lesion initiation and progression in vitro. Caries Res. (2014) 48(1):45–50. 10.1159/000351691 24247975

[22] Burkes S Mccleskey CS. The bacteriostatic activity of cerium, lanthanum, and thallium. J Bacteriol. (1947) 54(4):417–24. 10.1128/jb.54.4.417-424.1947 16561377 PMC526572

[23] Motewasselin N Hiller KA Cieplik F Kopp L Pfitzner A Pielnhofer F Cerium- and samarium-nitrate interaction and accumulation on human dentin. Arch Oral Biol. (2024) 167:106053. 10.1016/j.archoralbio.2024.106053 39029289

[24] Marciniak M Bałtrukiewicz Z. Effect of various doses of cerium on the serum level of ornithine-carbamyltransferase in rats. Acta Physiol Pol. (1981) 32(2):205–11. 7270221

[25] Akamp T Pohl S Scholz KJ Sigl P Rosendahl A Wölflick M In-vitro-cytotoxicity of cariostatic agents based on fluorides and lanthanide salts in L-929 fibroblasts. Clin Oral Investig. (2025) 29(7):366. 10.1007/s00784-025-06429-8 40593225 PMC12213984

[26] Exterkate RAM Crielaard W Ten Cate JM. Different response to amine fluoride by *Streptococcus mutans* and polymicrobial biofilms in a novel high-throughput active attachment model. Caries Res. (2010) 44(4):372–9. 10.1159/000316541 20668379

[27] Hahnel S. Die Eignung von Proteinmischungen als Speichelersatz für die Bakterielle in Vitro Adhäsion (thesis). Regensburg: University of Regensburg (2007).

[28] Muehler D Rupp CM Keceli S Brochhausen C Siegmund H Maisch T Insights into mechanisms of antimicrobial photodynamic action toward biofilms using phenalen-1-one derivatives as photosensitizers. Front Microbiol. (2020) 11:589364. 10.3389/fmicb.2020.589364 33193252 PMC7662152

[29] Cieplik F Wimmer F Muehler D Thurnheer T Belibasakis GN Hiller KA Phenalen-1-one-mediated antimicrobial photodynamic therapy and chlorhexidine applied to a novel caries biofilm model. Caries Res. (2018) 52(6):447–53. 10.1159/000487815 29617682

[30] Marsh PD Head DA Devine DA. Ecological approaches to oral biofilms: control without killing. Caries Res. (2015) 49(Suppl 1):46–54. 10.1159/000377732 25871418

[31] Cieplik F Kara E Muehler D Enax J Hiller KA Maisch T Antimicrobial efficacy of alternative compounds for use in oral care toward biofilms from caries-associated bacteria in vitro. Microbiologyopen. (2019) 8(4):e00695. 10.1002/mbo3.695 30051653 PMC6460264

[32] Hannig C Hannig M Rehmer O Braun G Hellwig E Al-Ahmad A. Fluorescence microscopic visualization and quantification of initial bacterial colonization on enamel in situ. Arch Oral Biol. (2007) 52(11):1048–56. 10.1016/j.archoralbio.2007.05.006 17603998

[33] Predoi D Iconaru SL Predoi MV Groza A Gaiaschi S Rokosz K Development of cerium-doped hydroxyapatite coatings with antimicrobial properties for biomedical applications. Coatings. (2020) 10(6):516. 10.3390/coatings10060516

[34] Kopp L Hiller KA Cieplik F Pfitzner A Pielnhofer F Höfler B Nitrates of cerium and samarium deposit on human enamel independently of a salivary pellicle. Front Oral Health. (2024) 5:1455924. 10.3389/froh.2024.1455924 39328894 PMC11425791

[35] Quintas V Prada-López I Donos N Suárez-Quintanilla D Tomás I. In situ neutralisation of the antibacterial effect of 0.2% chlorhexidine on salivary microbiota: quantification of substantivity. Arch Oral Biol. (2015) 60(8):1109–16. 10.1016/j.archoralbio.2015.04.002 26022118

[36] Toljanic JA Hagen JC Takahashi Y Shapiro RD. Evaluation of the substantivity of a chlorhexidine oral rinse in irradiated head and neck cancer patients. J Oral Maxillofac Surg. (1992) 50(10):1055–9. 10.1016/0278-2391(92)90490-Q 1527659

[37] Nisar A Iqbal S Ur Rehman A Mahmood M Younas A Hussain M Study of physico-mechanical and electrical properties of cerium doped hydroxyapatite for biomedical applications. Mater Chem Phys. (2023) 299:127511. 10.1016/j.matchemphys.2023.127511

[38] Cawthray JF Creagh AL Haynes CA Orvig C. Ion exchange in hydroxyapatite with lanthanides. Inorg Chem. (2015) 54(4):1440–5. 10.1021/ic502425e 25594577

[39] Kaur K Singh KJ Anand V Islam N Bhatia G Kalia N Lanthanide (=Ce, Pr, Nd and Tb) ions substitution at calcium sites of hydroxyl apatite nanoparticles as fluorescent bio probes: experimental and density functional theory study. Ceram Int. (2017) 43(13):10097–108. 10.1016/j.ceramint.2017.05.029

[40] Carniello V Peterson BW van der Mei HC Busscher HJ. Physico-chemistry from initial bacterial adhesion to surface-programmed biofilm growth. Adv Colloid Interface Sci. (2018) 261:1–14. 10.1016/j.cis.2018.10.005 30376953

[41] Poppolo Deus F Ouanounou A. Chlorhexidine in dentistry: pharmacology, uses, and adverse effects. Int Dent J. (2022) 72(3):269–77. 10.1016/j.identj.2022.01.005 35287956 PMC9275362

[42] Tam K Kinsinger N Ayala P Qi F Shi W Myung NV. Real-time monitoring of *Streptococcus mutans* biofilm formation using a quartz crystal microbalance. Caries Res. (2007) 41(6):474–83. 10.1159/000108321 17851235 PMC2820325

[43] Koo H Xiao J Klein MI Jeon JG. Exopolysaccharides produced by *Streptococcus mutans* glucosyltransferases modulate the establishment of microcolonies within multispecies biofilms. J Bacteriol. (2010) 192(12):3024–32. 10.1128/JB.01649-09 20233920 PMC2901689

[44] Bhatt L Chen L Guo J Klie RF Shi J Pesavento RP. Hydrolyzed ce(IV) salts limit sucrose-dependent biofilm formation by *Streptococcus mutans*. J Inorg Biochem. (2020) 206:110997. 10.1016/j.jinorgbio.2020.110997 32169780 PMC7233464

[45] Richards GA Brink AJ McIntosh R Steel HC Cockeran R. Investigation of biofilm formation on a charged intravenous catheter relative to that on a similar but uncharged catheter. Med Devices (Auckl). (2014) 7:219–24. 25018657 10.2147/MDER.S63449 PMC4074180

[46] Terada A Okuyama K Nishikawa M Tsuneda S Hosomi M. The effect of surface charge property on *Escherichia coli* initial adhesion and subsequent biofilm formation. Biotechnol Bioeng. (2012) 109(7):1745–54. 10.1002/bit.24429 22250009

[47] Jiang W Saxena A Song B Ward BB Beveridge TJ Myneni SCB. Elucidation of functional groups on gram-positive and gram-negative bacterial surfaces using infrared spectroscopy. Langmuir. (2004) 20(26):11433–42. 10.1021/la049043 15595767

[48] Gottenbos B Grijpma DW van der Mei HC Feijen J Busscher HJ. Antimicrobial effects of positively charged surfaces on adhering gram-positive and gram-negative bacteria. J Antimicrob Chemother. (2001) 48(1):7–13. 10.1093/jac/48.1.7 11418507

[49] Kurniawan A Yamamoto T Tsuchiya Y Morisaki H. Analysis of the ion adsorption–desorption characteristics of biofilm matrices. Microbes Environ. (2012) 27(4):399–406. 10.1264/jsme2.ME11339 22673305 PMC4103547

[50] Skrypnyk M Ananieva M Petrushanko T Neporada K Spivak M. Antimicrobial activity of chlorhexidine and cerium oxide nanoparticles composition. Acta Fac Med Naissensis. (2023) 40(4):479–88. 10.5937/afmnai40-41908

[51] Schubert A Wassmann T Holtappels M Kurbad O Krohn S Bürgers R. Predictability of microbial adhesion to dental materials by roughness parameters. Coatings. (2019) 9(7):456. 10.3390/coatings9070456

[52] Siegismund D Undisz A Germerodt S Schuster S Rettenmayr M. Quantification of the interaction between biomaterial surfaces and bacteria by 3-D modeling. Acta Biomater. (2014) 10(1):267–75. 10.1016/j.actbio.2013.09.016 24071002

